# Center-Related Determinants of VKA Anticoagulation Quality: A Prospective, Multicenter Evaluation

**DOI:** 10.1371/journal.pone.0144314

**Published:** 2015-12-04

**Authors:** Alberto Tosetto, Cesare Manotti, Francesco Marongiu, Italian Federation of Anticoagulation Clinics (FCSA) clinical quality study group

**Affiliations:** 1 Department of Hematology, S. Bortolo Hospital, Vicenza, Italy; 2 Hemostasis Center, Fidenza-Vaio Hospital, Fidenza, Italy; 3 Department of Internal Medicine, University of Cagliari, Cagliari, Italy; University of Bologna, ITALY

## Abstract

**Background:**

Center-specific TTR (c-TTR) is a measure reporting the mean patient TTR within an anticoagulation clinic describing the quality of anticoagulant monitoring offered by that clinic. c-TTR has a considerable between-center variation, but its determinants are poorly understood.

**Objectives:**

We aimed at evaluating which clinical, procedural or laboratory factors could be associated with c-TTR variability in a multicenter, observational cross-sectional study over a five-year period.

**Patients/Methods:**

Data from 832,204 individual patients followed for VKA therapy in 292 Centers affiliated with the Italian Federation of Anticoagulation Clinics (FCSA) were analyzed. c-TTR was computed based on the TTR of patients followed at each Center, and a mixed linear regression model was used for a predefined set of explanatory variables.

**Results:**

The Center next-visit interval ratio (the mean number of days after a visit with an INR outside the therapeutic range, divided by the days after a visit with an INR within the therapeutic range), the Center mean patient INR and the Center laboratory performance at EQA proficiency testing were the only variables that were independently associated with c-TTR (β-coefficients -17.32, 9.67, and -0.11, respectively; *r* ^*2*^ = 0.635).

**Conclusions:**

These findings suggest that c-TTR associates with proactive strategies aimed at keeping patients very close to their target INR with a prompt re-evaluation of those patients with under- or over-therapeutic INR.

## Introduction

Despite anticoagulant therapy with vitamin K antagonists (VKA) has been extensively used for more than fifty years, the relation between quality of anticoagulation control and adverse outcomes has been fully appreciated rather recently [1]. The definition of INR-based appropriate therapeutic ranges and the concept of time spent in the therapeutic range (TTR) [2, 3] have been instrumental in assessing the quality of VKA therapy. Several studies have validated patient TTR as a quantitative measure of the quality of anticoagulation control and as a predictor of bleeding and thromboembolic events [4–7].

Female gender, age <60 years, the presence of comorbidities, use of interfering drugs, smoking, and the nonwhite race associated with a low TTR in patients receiving VKA [8–10]. Other than these individual-related factors, TTR may be influenced by differences between of Anticoagulation Centers caring for the patients. This was first suggested by the wide fluctuations of Center TTR (c-TTR) that were observed in patients from different world regions in a sub-analysis of the ROCKET trial [11], and further confirmed in the RELY trial, with a c-TTR ranging from 44 to 77% [12].

Unfortunately, there is a paucity of data regarding the Center-related factors that may influence c-TTR. These data may be of considerable importance since (at variance with individual elements) appropriate interventions may actively change them and possibly improve c-TTR. For instance, variation in dose adjustment practice between each Center has been associated with c-TTR [9], as well as differences in mean target INR levels [13]. More importantly, a recent meta-analysis has concluded that the incidence of major bleeding events in patients on VKA is comparable to those taking new oral anticoagulants when the c-TTR is above 66% [14].

Since the quality of VKA therapy is worse in patients not enrolled in clinical trials [15], data abstracted from unselected cohorts would provide useful insights factors influencing the c-TTR in a real-life setting. Starting year 2009, the Italian Federation of Anticoagulant Clinics (FCSA) promoted the development of a prospective, multicenter observational study collecting data on the quality of VKA therapy in Italy. In this study, we report on the Center-related factors possibly influencing c-TTR in unselected patients over a five years interval.

## Materials and Methods

### Study design

The study has a multicenter, cross-sectional design. No intervention was planned or suggested to the participating Centers to improve their c-TTR over the observation years. Each participating Center received a yearly report detailing its performance (in terms of TTR values) against the mean TTR of the other participating FCSA Centers, without any correlation with other study variables.

### Center-related data

For mandatory accreditation, all FCSA Centers are required to provide detailed information about their structure every two years, and to participate in external quality assessment (EQA) laboratory proficiency twice a year. A database containing the Centers’ data and proficiency tests is regularly maintained by FCSA. From the 2008 FCSA database, information was abstracted for each Center according to three domains: a) allocated resources (number of physicians and nurses workers providing direct patient assistance at the Center); b) presence of formal protocols for VKA therapy management (documented procedures for monitoring patient compliance and lost to follow-up, VKA over- and under-dosing, and for the tracking of adverse events); c) patient education (availability of patient educational material or regular meetings with patients). Collection of this information was planned a priori by the study protocol as it was felt to influence the Center performance; no missing data was present for these variables. All the variables mentioned above were entered into the analysis. For the mandatory EQA proficiency, a set of three lyophilized human plasmas, each one having a different INR, is sent twice a year to all FCSA Centers. For each plasma sample, the deviation of the measured INR from the mean INR of all participating Centers (*z-*score) was centrally computed. A *z-*score of zero indicates optimal performance, whereas a z-score of 1 or 2 indicates deviation from the mean INR equal to one or two standard deviations, respectively.

### Patient-related data

Starting January 1^st^ 2010, all FCSA Centers were invited to participate in this observational study, by annually providing the electronic records of all visits to patients receiving VKA therapy in the preceding year; data were included in this study if a participating Center provided data for at least one year. All patient records were anonymized and de-identified prior to analysis. The full dataset, therefore, comprises data from January 1^st^, 2009 until December 31^st^, 2013 when the database was locked. Each record contained information of visit time, patient INR and therapeutic interval (i.e., the lower and upper INR boundaries considered as acceptable in the patient), anticoagulant type (either warfarin or acenocoumarol) and weekly dose. Also, Centers provided individual baseline data (date of birth, gender, indication for and date of inception of VKA therapy). All participating Centers used a commercial computerized decision support system, but the final decision about dose prescription was left at the discretion of Centers’ physician.

### Data analysis

From the cumulative, five-year database of all patient observations we computed the annual TTR in all subjects according to the Rosendaal method [2], when the time between two INR determinations was lower than 60 days using linear interpolation. The c-TTR was then computed as the annual average of the TTR of patients followed by that Center. Finally, we calculated the within Center next visit interval (NVI) ratio as the ratio of days between visits when the INR was below or above range divided by days between visits when the INR was in the therapeutic range. This index is 1 when there is no difference between the prescribed time interval to next visit in patients with satisfactory or unsatisfactory INR; values <1 indicate the Center attitude for shortened intervals between visits in case of inadequate INR. Thus, the NVI ratio describes the Center’s attitude toward anticipating the next control in case of non-therapeutic INR.

Since the same FCSA Center may contribute up to five observations in the dataset (if participating every year from year 2009 to 2013), and the within-Center variability is expected to be less than the between-Center one, we used a linear mixed model to evaluate the relationship between each year c-TTR and the explanatory, independent variables and the Center baseline data [16, 17]. In this model, within-Center variances are separately accounted using a random intercept linear model having the same coefficient for each explanatory variable with separate intercepts for each Center. By eliminating the within-Center variance, this method is more robust to detect even small determinants of between-Center variability [16]. Variables were entered in the model as dummy variables when dichotomous or as quartiles when discrete (e.g., the number of allocated physicians). To evaluate the relationship between Center TTR and EQA proficiency performance, we computed for each Center the annually averaged total of absolute *z-*scores. For instance, if a Center had *z-*scores -0.47, -0.06, -0.52 at the first yearly round and 0.12, 0.64, 0.41 at the second one, then its annual averaged total deviation would be (1.05+1.17)/2 = 1.11. The annual averaged absolute deviation was, therefore, tested in the linear mixed model. The relation between explanatory variables and TTR was first tested on all variables previously described and belonging to the three domains; variables significant at the p = 0.05 level were subsequently evaluated in a multivariate model. The Snijders/Bosker r square was used to assess the goodness of the model fit. All analyzes were carried out using the Stata software package [18].

## Results

During the five-year interval, data were collected from a total of 292 FCSA Centers on 832 204 individual patients followed for VKA therapy; Table 1 reports participating Centers and patients for each study year. The median patient age was 77 years (interquartile range 69–83); 51.3% were female. The median time between two visits was 17 days (interquartile range 10–22 days). Atrial fibrillation was the leading indication for VKA therapy (68.7%), followed by venous thromboembolism (15.6%) and prosthetic valve (10.9%); 4.8% received VKA for other indications. The participation rate of FCSA Centers to the survey ranged from 52.7% (year 2009) to 57.4% (year 2014).

**Table 1:** Characteristics of the patients included during the five years study.

	Year				
	2009	2010	2011	2012	2013
Participating / Non participating FCSA Centers in the year	156/296	201/397	178/409	233/424	252/439
Evaluated patients, n	163890	206117	186906	254081	308178
Male gender, %	48.1	48.0	48.5	48.2	48.9
Median patient age, years (IQL range*)	76 (68–82)	76 (69–82)	77 (69–83)	77 (69–83)	78 (70–84)
Median number of visits per patient (IQL range*)	16 (12–21)	16 (12–20)	16 (12–21)	17 (12–21)	16 (12–20)
Median patient INR (IQL range*)	2.37 (1.99–2.82)	2.39 (2.00–2.83)	2.38 (2.00–2.83)	2.39 (2.00–2.83)	2.39 (2.01–2.82)
Reason for VKA					
Atrial Fibrillation %, (n)	67.6% (93249)	67.6% (118305)	68.5% (102985)	68.6% (138849)	68.3% (149328)
VTE%, (n)	15.4% (21269)	15.3% (26736)	15.4% (23194)	15.6% (31520)	15.5% (33816)
Valvular prosthesis %, (n)	13.0% (17967)	12.0% (21032)	11.6% (17401)	10.6% (21383)	10.1% (22041)
Other indications %, (n)	4.0% (5466)	5.1% (8885)	4.5% (6777)	5.3% (10779)	6.1% (13319)

**Fig 1:**
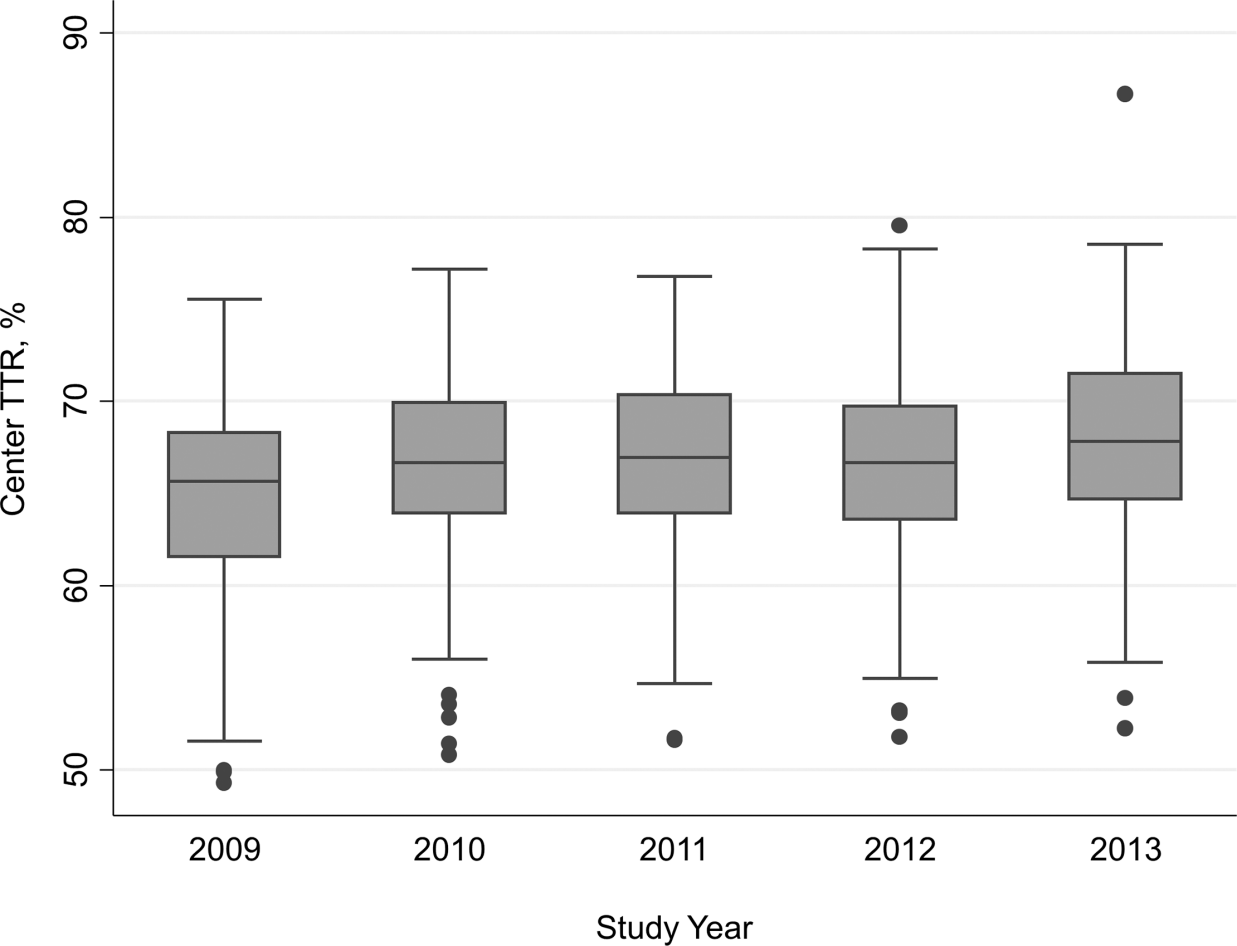
Boxplot of Center TTR by study year, showing the overall increase of anticoagulation quality over the years.

**Fig 2:**
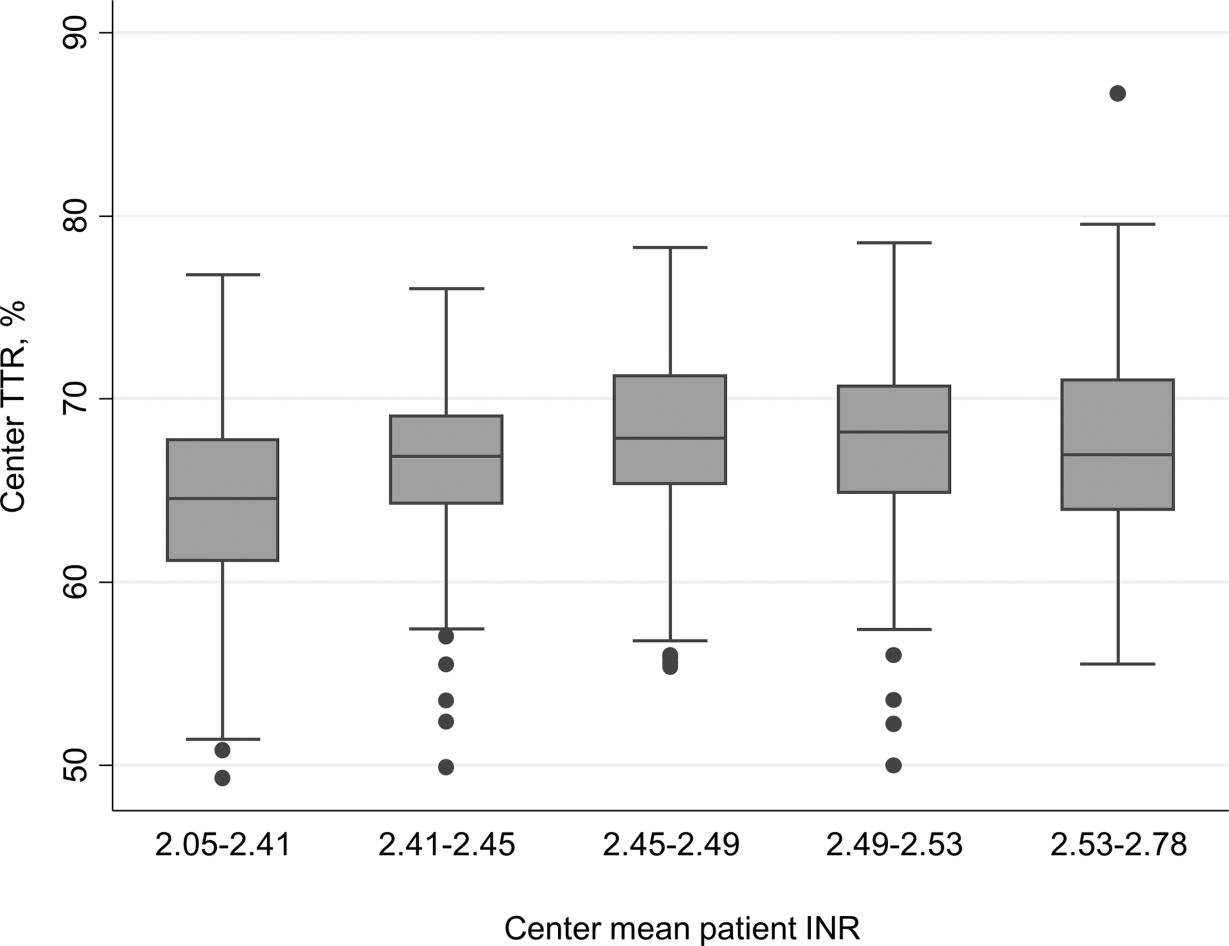
Boxplot of Center TTR by quintiles of Center mean patient INR, showing an inverse U-shaped relationship between mean patient INR and TTR.

**Fig 3:**
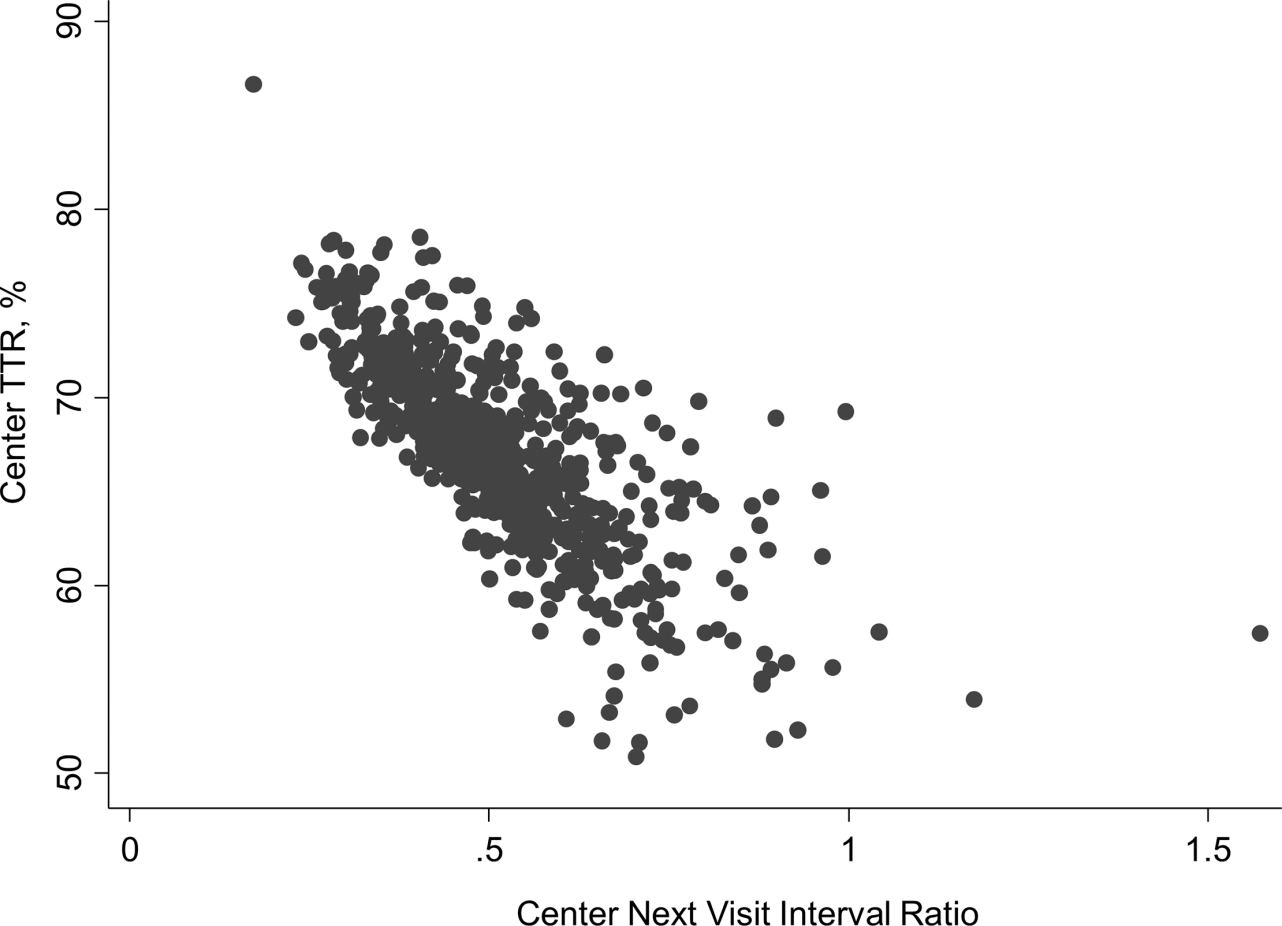
Relationship between Center TTR and Center Next Visit Interval (NVI) ratio, which is defined as the ratio of days between visits when the INR was below or above range divided by days between visits when the INR was in the therapeutic range.

**Fig 4:**
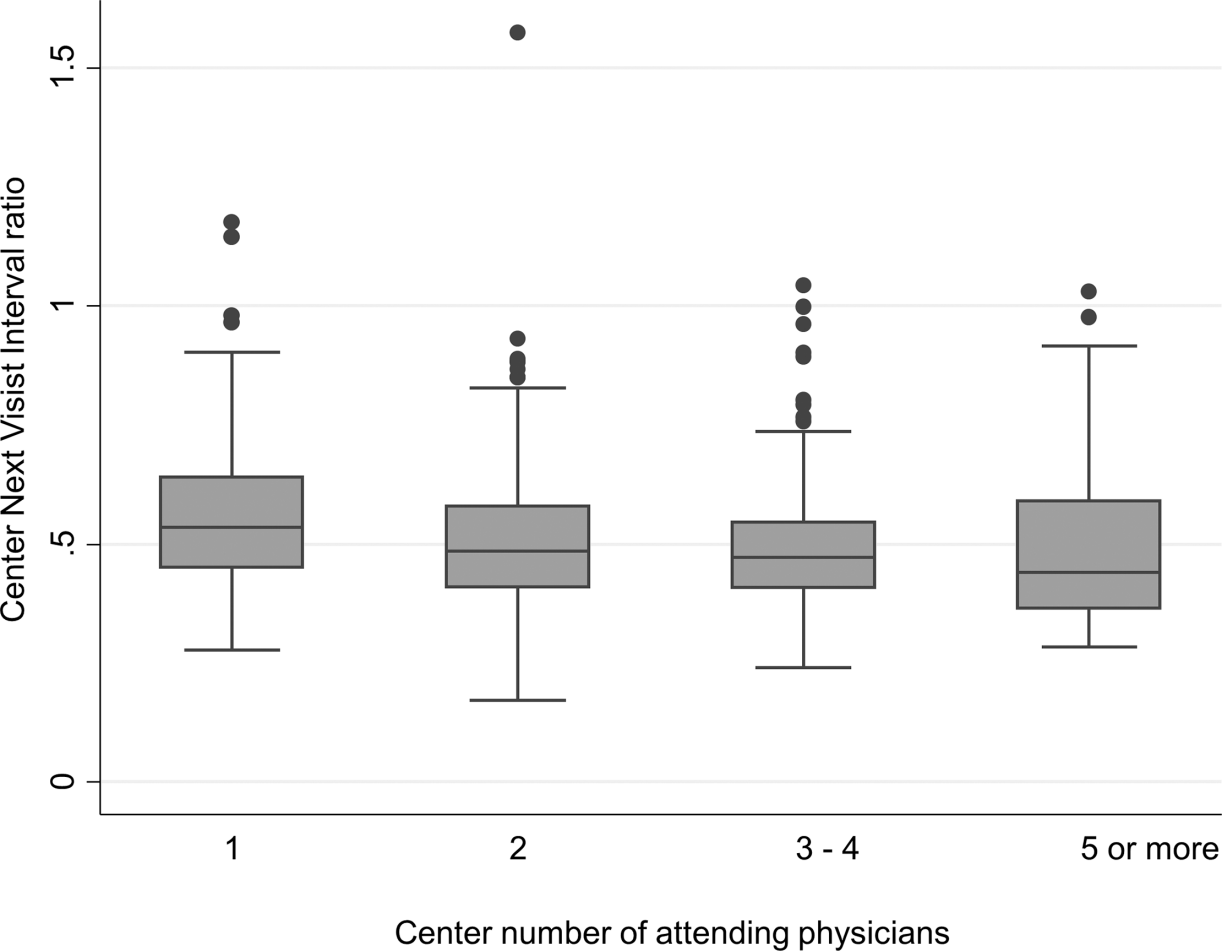
Boxplot of Center Next Visit Interval (NVI) ratio by number of physicians attending the Anticoagulation Clinic, showing an inverse relationship (p<0.0001 for trend).

* Interquantile range

c-TTR showed a considerable variability and significantly increased over the 5-year observation time **(** Fig 1 **),** rising from 64.8% (range 49.2–75.5%) in 2009 up to 67.9% (range 52.2–86.6%) in 2013 (p for trend <0.001). The percentage of Centers with a TTR above 60% was 83.9 in 2009 and 93.6 in 2013 (p = 0.025). Table 2 reports the results of the univariate analysis that was performed on the variables belonging to the three Center domains *a priori* considered as possibly relevant. Having 3 or more physicians caring for the patients was associated with an improvement of c-TTR, together with the availability of written protocols for the management of nontherapeutic INR at the site. In addition to Center-related variables, univariate analysis showed that a powerful determinant of c-TTR was the Center mean patient INR, broadly reflecting the Center attitude to keep patients close to the target therapeutic INR. A higher Center TTR was observed particularly when the Center mean INR was between 2.45 to 2.53 (the middle quintile), and decreasing at the lowest or highest quintiles (Fig 2). The median Center Next Visit Interval ratio was 0.48 (range 0.27–0.97; interquartile range 0.40–0.58). This means that, as median attitude, Centers tend to halve the days to next control when an INR is under- or over-therapeutic, resulting in a next visit interval of about 8 days instead of 17 in such circumstances. The Center Next Visit Interval ratio was very strongly associated with c-TTR, as could also be graphically appreciated in Fig 3 that shows the unadjusted correlation. There was a significant association between the Center NVI ratio and the number of physicians available at the Center (p for trend 0.0001; Fig 4). Since some patient characteristics may also influence to some degree the c-TTR, we also modeled for the proportion of male/female patients, having atrial fibrillation as indication for treatment, or with time from inception of VKA treatment <3 months and the mean Center’ patients age. A slight improvement of c-TTR was observed only for those Centers having an increased proportion of patients with atrial fibrillation and with time from inception of VKA treatment ≥3 months. We subsequently entered into the multivariate mixed linear model all the previously identified c-TTR determinants having a p<0.05 at univariate analysis. Only the Center NVI ratio, Center mean patient INR and the annual averaged total of absolute *z-*scores (i.e., the laboratory performance at EQA proficiency testing) remained independently associated with c-TTR (Table 3). These findings were not substantially influenced by adding to the multivariate model patient-specific covariates (proportion of patients with atrial fibrillation and with time from inception of VKA treatment ≥3 months).The Snijders/Bosker *r* ^*2*^ for the full multivariate model was 0.646, while for the model not including patient-specific covariates was 0.635.

**Table 2:** Univariate analysis of determinants of Center TTR, using multilevel mixed regression to adjust for repeated measures for each Center.

Variable	Regression β-coefficient	95% CI
Allocated resources *		
Two physicians (vs. 1)	1.14	-0.46–2.75
3–4 physicians (vs. 1)	2.02	0.32–3.71
5 or more physicians (vs. 1)	2.20	-0.35–4.76
Two nurses (vs. 1)	-3.11	-6.05 –-0.22
3 or more nurses (vs. 1)	-3.35	-6.32 –-0.37
VKA management protocols#		
Compliance monitoring (yes vs. no)	-0.63	-1.93–0.66
Written protocol for non-therapeutic INR management (yes vs. no)	1.40	0.10–2.91
Lost to follow-up patient tracing (yes vs. no)	-0.25	-1.47–0.97
Adverse events tracing (yes vs. no)	-0.76	-2.21–0.68
Patient education provided through#		
Physician, face to face (yes vs. no)	0.45	-2.24–3.14
Written material (yes vs. no)	0.88	-0.39–2.15
Periodic patients’ meetings (yes vs. no)	0.38	-0.87–1.64
Laboratory performance at EQA proficiency testing#	-0.16	-0.28 –-0.03
Center mean patient INR#	14.8	11.37–18.26
Center NVI ratio#	-18.15	-19.57 –-16.74
Center mean patient age#	0.04	-0.03–0.12
Center male proportion#	2.52	-1.77–6.82
Center proportion of patients with atrial fibrillation#	1.54	0.39–2.68
Center proportion of patients <3 months from VKA inception#	-2.72	-3.79 –-1.65

* adjusted for study year and number of Center’s patients; # adjusted for study year

**Table 3:** Multivariate analysis of determinants of Center TTR using multilevel mixed regression to adjust for repeated measures for each Center, after backward elimination. All results are adjusted for study year.

Variable	Regression β-coefficient	95% CI
Laboratory performance at EQA proficiency testing	-0.11	-0.21 –-0.001
Center Mean patient INR	9.91	7.19–12.64
Center NVI Ratio	-25.06	-27.84 –-22.29
Center proportion of patients with atrial fibrillation	1.61	0.76–2.45
Center proportion of patients <3 months from VKA inception	-3.30	-6.00 –-0.59

## Discussion

The time spent in the therapeutic range (TTR) is a measure of the quality of management of anticoagulation therapy in patients receiving VKA therapy that correlates with adverse outcomes. Previous meta-analyses have shown that TTR is generally better in patients followed by anticoagulation clinics than in community settings (64.0 vs. 51.0) [15, 19], but there remains a need for improvement of VKA management as this translates into a burden of potentially avoidable hemorrhagic and thrombotic events [20–22]. The variability of c-TTR (i.e., the mean TTR of patients followed by a particular anticoagulation clinic) is also particularly striking. In a recent meta-analysis, the between-Center TTR variability ranged from 40 to 78% [19] and even in randomized controlled trials the between Centers variability ranged from 44% to 77% [12]. There is, therefore, a definite need for evaluating determinants of quality of anticoagulation clinics performance, and to improve on it by collaborative efforts. For this reason, the Italian Federation of Anticoagulation Clinics (FCSA) endorsed a program of VKA treatment quality monitoring since the year 2009.

In this study, the median c-TTR was 67.9% in the year 2013. A recent meta-analysis and recent clinical trials comparing VKA with direct oral anticoagulants found that median c-TTR ranges from 58.0 to 68.4% [23–26]. Interestingly, in our study there was a clear trend toward an increase in c-TTR over the five-year study period, suggesting that participating in an external assessment of therapy quality may improve the Center performance. The present study had a purely observational design, and no suggestion were provided to participating Centers to improve the quality of their mean TTR. Since each Center received a report contrasting their c-TTR with the average c-TTR of the other participants, it is nonetheless plausible that less performing Centers may have taken some steps toward improving their quality.

The primary determinants of c-TTR were primarily the Center NVI ratio, the Center mean patient INR and, to a lesser extent, the quality of laboratory INR measurement. These three factors explain about 63.5% of the total TTR variability among FCSA Centers in the years 2009–2013. To put this data in perspective, a recent meta-analysis has shown that 6.9% improvement in the TTR may reduce major hemorrhage by one event per 100 patient-years of treatment [27]. In our study, shifting the Center mean patient INR from the first quintile to the middle one could lead to an improvement of c-TTR up to 4% (Fig 2); the c-TTR change from the first to last quintile of Center NVI ratio is more than 11% (Fig 3). Therefore, it is likely that the improvement on these two variables may have a profound impact on the clinical management of patients taking VKA therapy, possibly reducing adverse outcomes of the treatment.

Prompt repeat testing after an out-of-range INR has been already shown to correlate with lower TTR [28]. In this study, we show that a significant improvement in Center performance is attained by reducing the time to the next control to 30% of the average interval, corresponding therefore to no more than one week. Adherence to a predefined warfarin dose adjustment practice was found to correlate with improved c-TTR in a sub-analysis of the RELY trial [9]; such an adherence explained 87% of the between Centers TTR variation. The RELY study protocol suggested a further visit to be planned within one week in the case of abnormal INR. It is, therefore, possible that most of the benefit associated with adherence to that proposed protocol was related to the shortened time to next visit. Adoption of a protocol suggesting reduced time to next visit in case of abnormal INR has also been demonstrated by a pilot study in Canada [29]. It is also worthwhile noting that the Center NVI ratio was also significantly associated in our study with the number of physicians attending VKA patients at the Clinic. The association between the number of doctors and availability of written protocols was found to be associated with improved c-TTR at univariate analysis, but both these factors were trumped by Center NVI ratio at multivariate analysis. We may, therefore, conclude that adherence to written protocols by an adequate staff is a critical factor for c-TTR and that this is primarily mediated by a reduction of Center NVI ratio.

We were able to confirm the relevance of standardizing INR targets to prescribed ones [13]. Centers having a mean patient INR close the target also have the higher c-TTR, whereas lower c-TTR were observed particularly for Centers targeting patients to lower INRs (Fig 2). When interpreting these findings, some caution should be excercized because the Center mean patient INR could have some degree of collinearity with c-TTR, and hence estimates may be overoptimistic. The relationship between the laboratory performances at EQA proficiency testing and c-TTR is quantitatively less than for the two previous variables, but it is nonetheless an important proof-of-concept. Since the median annual absolute *z-*score (over three samples) was 2.3, with an interquartile range from 1.6 to 3.2, only a very scarce laboratory performance could result in a clinically relevant reduction of c-TTR. Nonetheless, this Center characteristic remained independently also associated at multivariate analysis, suggesting that a good laboratory performance could be considered as a marker of good clinical practice as well.

The study has both strengths and limitations. We were able to follow-up a vast amount of unselected patients, totaling more than eight hundred thousand over the five years’ observation and constituting the largest VKA population ever studied. A consistent algorithm was used to compute TTR over the observation time, and multilevel analysis employed to account for within-Center effects. The lack of a homogeneous protocol to manage patients with under or over therapeutic INR between FCSA Centers is a study weakness, despite FCSA published guidelines for the management of VKA therapy [30, 31]. Furthermore, we relied on self-reported Center characteristics to evaluate their association with Center TTR, and this may have introduced an information bias. A further study limitation is the lack of a shared between-Centers protocol for assessment of adverse outcomes during warfarin therapy.

In conclusion, this national, multicenter, prospective cohort study demonstrates that the quality of therapy offered by Anticoagulation Clinics is associated with the capability of keeping patients near their target INR and with prompt re-evaluation of those patients with nontherapeutic INR. This competence is broadly dependent on the number of attending physicians and availability of written protocols. These novel findings explain >60% of the between-Centers variabily of c-TTR and are relevant to optimize clinics performance. We urge Anticoagulation Clinics to develop strategies and protocols aimed at developing a proactive attitude and improving these critical factors.

## Supporting Information

S1 Appendix List of participating Centers. (DOCX)

S2 Appendix Study Dataset. (ZIP)
