# Supplementary material for: Center-Related Determinants of VKA Anticoagulation Quality: A Prospective, Multicenter Evaluation
Source: PLoS One. 2015 Dec 4;10(12):e0144314. doi: 10.1371/journal.pone.0144314 (PMC4670074; doi:10.1371/journal.pone.0144314)
Supplement: S1 Appendix — (DOCX) [file pone.0144314.s001.docx]

**S1 Appendix. List of FCSA Centers and participants to the clinical quality study group**

N. 1, S. Giovanni Rotondo (Dr. Alicino Giovanni); N. 4, Ferrara (Dr. Maria Luisa Serino); N. 8, Borgo Val Di Taro (Dr. Guarneri Daniele); N. 13, Roma (Dr. Raimondo De Cristofaro); N. 18, Vallo Della Lucania (Dr. Giulio Feola); N. 22, Alessandria (Dr. Laura Contino); N. 28, Brescia (Dr. Giuliana Martini); N. 31, Merate (Dr. Erba Nicoletta); N. 34, Como (Dr. Vaghi Umberto); N. 40, Acquaviva Delle Fonti (Dr. Lucarelli Giacomo); N. 44, Parma (Dr. Quintavalla Roberto); N. 46, Torino (Dr. Ciardiello Aurora); N. 48, Oristano (Dr. Mulas Giuseppina); N. 56, Napoli (Dr. Lupone Maria Rosaria); N. 59, Sassari (Dr. Piseddu Gavino); N. 61, Gallipoli (Dr. Ria Luigi); N. 64, Vicenza (Dr. Tosetto Alberto); N. 71, Padova (Dr. Pengo Vittorio); N. 74, Lecco (Dr. Erba Nicoletta); N. 76, Gavardo (Dr. Tani Dr. Marco); N. 78, Seriate (Dr. Coffetti Nadia); N. 80, Milano (Dr. Marco Moia); N. 83, Latina (Dr. Carlo Ciabatta); N. 86, Cosenza (Dr. Rossi Vincenza); N. 89, Trento (Dr. Gosetti Grazia); N. 94, Catanzaro (Dr. Rita Santoro); N. 97, Milano (Dr. Russo Umberto); N. 99, Siena (Dr. Cappelli Roberto); N. 110, Palermo (Dr. Girolamo Spilotros); N. 116, Alzano Lombardo (Dr. Coffetti Nadia); N. 123, Savona (Dr. Contessini Michela); N. 125, Rho (Dr. Mutinelli Maria Rosa); N. 127, Manerbio (Dr. Manisco Luigia); N. 130, Vimercate (Dr. La Rosa Lucia); N. 134, Vibo Valentia (Dr. Scarmozzino Vincenzo); N. 138, Milano (Prof. Marco Cattaneo); N. 141, Cagliari (Dr. Porcu Alessandro); N. 143, Reggio Emilia (Dr. Nicolini Alberto); N. 146, Lanciano (Dr. Corti Itala); N. 149, Benevento (Dr. Domenico Parente); N. 153, Verona (Dr. Poli Giovanni); N. 156, Lecce (Dr. Campobasso Maria); N. 160, Torino (Dr. Martini Aurelio); N. 165, Orbassano (Dr. Calabrese Anna Maria); N. 168, Treviglio (Dr. Agostinelli Enrica); N. 171, Cantu' (Dr. Serricchio Giuseppina); N. 175, Legnano (Dr. Prof. Antonino Mazzone); N. 177, Imola (Dr. Conti Marinella); N. 182, Sapri (Dr. Doddato Angelo); N. 187, Nuoro (Dr. Maria Antonia Contu); N. 190, Acqui Terme (Dr. Pedrazzi Enrico); N. 194, Mercato S. Severino (Dr. Pennasilico Francesco); N. 197, Piombino (Dr. Fabio Pini); N. 203, L'Aquila (Dr. Orecchioni Assunta); N. 209, Albano (Dr. Massimo Sabatini); N. 212, Firenze - Bagno A Ripoli (Dr. Massimo Feliciangeli); N. 216, Cles (Dr. Orsoni Giordana); N. 223, Bergamo (Dr. Roberto Caccia); N. 225, Feltre (Dr. Liva); N. 227, Faenza (Dr. Bucherini Eugenio); N. 229, Penne (Dr. Ursitti Gesualdo); N. 236, Castellammare Di Stabia (Dr. Imbimbo Veneranda); N. 239, Galatina (Dr. Mangione Catello); N. 243, Andria (Dr. Suriano Luciano); N. 249, Reggio Calabria (Dr. Ruggeri Albarosa); N. 251, Potenza (Dr. Maria Antonietta Rizzo); N. 259, Torino (Dr. P.Milillo); N. 263, Asola (Dr. Gabriella Collina); N. 269, Novara (Dr. Gianluca Gaidano); N. 273, Vittorio Veneto (Dr. Zanatta Nello); N. 275, Genova (Dr. Marco Mori); N. 278, Pisa (Dr. Paolo Chiarugi); N. 281, Pisa (Dr. Lucia Ruocco); N. 283, Ostuni (Dr. Paolo Ciola); N. 285, Latina (Dr. Redi Roberta); N. 287, Mantova (Dr. Carlo Bonfanti); N. 291, Pieve Di Coriano (Dr. Semerari Angelo); N. 295, Arco (Dr. Vilma Tonini); N. 301, Chieti (Dr. Patrizia Di Gregorio); N. 310, Tagliacozzo (Dr. Santini M.Simonetta); N. 312, Roma (Dr. Laura Di Prospero); N. 315, Popoli (Dr. Raffaello Consorte); N. 319, Avezzano (Dr. Alessandra Pizzoferrato); N. 322, Milano (Dr. Vittorio Agape); N. 330, Roma (Dr. Luigi Amati); N. 333, La Spezia (Dr. Franco Giuseppe Sbarbaro); N. 337, Moncalieri (Dr. Antonio Insana); N. 340, Vobarno (Dr. Letizia Cesari); N. 344, Roma (Dr. M. Ersilia Lamioni); N. 346, Bussolengo (Dr. Caputo Marco); N. 352, Agropoli (Dr. Pasquale Carrato); N. 354, Albenga (Dr. Giorgio Alberti); N. 356, Cairo Montenotte (Dr. A. Marchetti); N. 359, Stradella (Dr. Pietropaolo Bianchi); N. 362, Paola (Dr. Antonio Rende); N. 367, Cuorgne' (Dr. Alessandro Confalonieri); N. 370, Roma (Dr. Claudio Vasselli); N. 373, Lodi (Dr. Marco D'Agostino); N. 376, Squinzano (Dr. Maria Novella Di Pierro); N. 385, San Gavino Monreale (Dr. Ronchi Francesco); N. 389, Monterotondo (Dr. Ileana Galani); N. 392, Brescia (Dr. Simona Pedrini); N. 395, Palermo (Dr. Tommaso Gristina); N. 400, Perugia (Dr. Eliana Santucci); N. 402, Torino (Dr. Maurizio Molinatti); N. 404, Verbania Pallanza (Dr. Maria Chiara Rolla); N. 406, Monza (Dr. Chiara Bersano); N. 408, Borgo San Lorenzo (Dr. Franco Rosichini); N. 413, Bari (Dr. Eufemia Savino); N. 419, Ruvo Di Puglia (Dr. Vincenza Marlend Agatino); N. 422, Castelnovo In Monti (Dr. Vincenzo Insegnante); N. 425, Casale Monferrato (Dr. Vaniglia Francesca); N. 427, Foggia (Dr. Silvana F. Capalbo); N. 431, Matera (Dr. Tonino Valentino); N. 438, Roma (Dr. Milano Cesare); N. 440, Roma (Dr. Lentini Giuseppe); N. 443, San Daniele Del Friuli (Dr. Mazzanti Gabriella); N. 447, Nocera Inferiore (Dr. Rescigno Giuseppe); N. 451, Sora (Dr. Reggio Raffaele); N. 454, Salerno (Dr. Giuseppe Capuano); N. 459, Cosenza (Dr. Maria Carmela Bilotta); N. 461, Barletta (Dr. Eugenio Peres); N. 463, Napoli (Dr. Pennino Domenico); N. 465, Bari (Dr. Gesario Antonia); N. 467, Ascoli Piceno (Dr. Nardi Gilda); N. 470, Castellana Grotte (Dr. Anna Maria Sonnante); N. 474, Castiglione Delle Stiviere (Dr. Adinolfi Gabriele); N. 478, Udine (Dr. Barillari Giovanni); N. 480, Mesagne (Dr. Miccoli Giancarlo); N. 484, Pavia (Dr. Franco Piovella); N. 486, Modica (Dr. Enzo Fidone); N. 489, Vasto (Dr. Emilio Bolognese); N. 491, Bozzolo (Dr. Antonio Pernigotti); N. 494, Altamura (Dr. Giuseppe Ninivaggi); N. 496, Gagliano Del Capo (Dr. Aniello Carbone); N. 498, Roma (Dr. Marcella Ferrari); N. 500, Roma (Dr. Odoardo M. Olimpieri); N. 503, Roma (Dr. Lorena Silvestri); N. 507, Scorrano (Dr. Mario Schiavoni); N. 511, Cassino (Dr. Fausto Marrocco); N. 514, Triggiano (Dr. Triggiani Rita); N. 519, Ostuni (Dr. Paolo Ciola); N. 522, Gioia Del Colle (Dr. Anna Rina Iacobelli); N. 525, Gioia Tauro (Dr. Edoardo Macino); N. 528, Tricase (Dr. Luciano Abbruzzese).
